# The effects of crop diversity and crop type on biological diversity in agricultural landscapes: a systematic review protocol

**DOI:** 10.12688/wellcomeopenres.15343.2

**Published:** 2020-07-27

**Authors:** Cami Moss, Martin Lukac, Francesca Harris, Charlotte L. Outhwaite, Pauline F.D. Scheelbeek, Rosemary Green, Fernanda Morales Berstein, Alan D. Dangour

**Affiliations:** 1 Faculty of Epidemiology and Population Health, London School of Hygiene & Tropical Medicine, London, WC1E 7HT, UK; 2 Centre on Climate Change and Planetary Health, London School of Hygiene & Tropical Medicine, London, WC1E 7HT, UK; 3 School of Agriculture, Policy and Development, University of Reading, Reading, RG6 6AR, UK; 4 Centre for Biodiversity and Environment Research, University College London, London, WC1E 6BT, UK

**Keywords:** crop diversity, intercropping, crop rotation, agricultural management, biodiversity, species richness, abundance

## Abstract

Agricultural intensification is a well-known driver of biodiversity loss. Crop diversity and its changes over space and time drive land use intensity and impact biodiversity of agricultural landscapes, while meeting the growing demand for human food and nutrition resources. Loss of biodiversity in agricultural landscapes reduces primary productivity and soil health and erodes a range of other ecosystem services. At present, while having partial understanding of many processes, we lack a general synthesis of our knowledge of the links between crop diversity and biodiversity. We will therefore conduct a systematic review by searching multiple agriculture, ecology and environmental science databases (e.g. Web of Science, Geobase, Agris, AGRICOLA, GreenFILE) to identify studies reporting the impacts of crop diversity and crop type on the biological diversity of fauna and flora in agricultural landscapes. Response variables will include metrics of species richness, abundance, assemblage, community composition and species rarity. Screening, data coding and data extraction will be carried out by one researcher and a subset will be independently carried out by a second researcher for quality control. Study quality and risk of bias will be assessed. Evidence will first be mapped to species/taxa then assessed for further narrative or statistical synthesis based on comparability of results and likely robustness. Gaps in the evidence base will also be identified with a view toward future research and policy directions for nutrition, food systems and ecology.

## 1. Background

Land use and land use intensity are recognised as the primary drivers of biodiversity loss in agricultural landscapes. Selection of crop types – defined as major categories of intensively-grown domesticated plants – and related management and production cycles determine the intensity of agricultural management ^1^. Intensification factors that have been well researched in relation to biodiversity include landscape heterogeneity ^2– 4^, use of pesticides ^5– 7^ and fertilisers ^8– 10^, and ploughing ^11, 12^. Crop diversification (i.e. the addition of new crops or cropping systems on a farm) has been proposed as a management practice that may reduce some of the environmental impacts of modern farming related to fertiliser and pesticide use and conventional tillage. Therefore, crop diversity may mitigate some food production-biodiversity trade-offs ^13^– namely, that conventional high-input intensification of agricultural land use reduces conversion of natural habitats but also decreases biodiversity ^14, 15^.

Crop diversity has spatial and temporal dimensions. Practices such as mixed cropping or intercropping, and growing diverse crops at landscape scale, characterise agricultural diversity in space. Rotation of crops provides agricultural diversity over time. Increased crop diversity over both space and time is associated with improved soil health, pest control, decreased erosion, and increased nutrient cycling ^16^. However, relationships between crop diversity and the biodiversity of flora and fauna are less clear and synthesis of the current literature may provide useful insights to help inform the debate on land use trade-offs related to future food production.

Crop types are also known to have impacts on biodiversity that are independent of crop diversity benefits, for example, that of wheat on soil microbial diversity ^17^ or fruit orchards on bird abundance ^18^. Evidence of these relationships has not yet been mapped or synthesised. Understanding the relationships between crop type and biodiversity – even if mediated by agricultural intensity – may help support the sustainable increase of agricultural production in coming decades through crop selection processes optimised for human and ecological well-being. For purposes of this study, crops are defined as plants or trees cultivated for human and animal use or consumption including food, feed, selected cover crops, fibres, fuels, and grasslands/herbage for pasture. Whilst within-species genetic diversity of crops, including wild relatives, is very important to future breeding efforts due to potential benefits such as nutritional content or resilience to environmental stress, it is beyond the scope of this review and will not be considered.

Biodiversity is complex and no single metric can assess its multiple dimensions including genetic, species, functional and ecosystem diversity, as it exists over time and space ^19^. Nevertheless, commonly used metrics include species extinction and extinction risk, species richness (the number of species in a grid), abundance (the number of individuals per species), and community composition or assemblage of species in a given grid. Rare species richness and relative species rarity are also thought to capture aspects of biodiversity related to functional and phylogenetic diversity ^20, 21^. These measurements are practical and individually capture important, if incomplete, dimensions of biodiversity; consequently, they are also the most used in the environmental sciences. This is the first systematic literature review to examine and synthesise literature on the relationship between crop diversity and crop type on common metrics of biodiversity.

## 2. Aim and objectives

The aim of this review is to answer the primary research question: “What are the effects of spatial and temporal crop diversity and of individual crop type on the biological diversity of fauna and flora in agricultural landscapes?”

Secondary questions to be answered by this study include:

- Which species or taxonomic groups are most affected by crop diversity?
- Which crop diversification practices have the strongest impacts on biodiversity?

The study objectives are:

- To identify, assess and summarise studies that have estimated the impacts of crop diversity and crop type on biodiversity among flora and fauna.
- To map and synthesise evidence of the impacts of spatial and temporal crop diversity on biodiversity.
- To identify trends in the response of biodiversity to crop diversity across different taxonomic groups or biomes.
- To map evidence of the impacts of crop type on bio-diversity.
- To highlight research gaps.

## 3. Methods

### 3.1. Search strategy

Due to the transdisciplinary nature of the research, multiple databases covering the fields of environment and ecological sciences and agriculture will be searched, namely: 1) Web of Science Biological Abstracts, Reports, Reviews, and Meetings (BIOSIS) Citation Index (Clarivate Analytics), 2) Web of Science, Science Citation Index (Clarivate Analytics), 3) Commonwealth Agricultural Bureaux (CAB) Abstracts (Ovid), 4) Geobase (Ovid), 5) International System for Agricultural Science and Technology (AGRIS) (UN Food & Agriculture Organisation), 6) GreenFILE (Ebsco), 7) AGRICOLA (AGRICultural OnLine Access) (USDA National Agricultural Library), 8) Northern Light (Ovid), 9) Open Grey (INIST-CNRS), and 10) Dissertations & Theses Global (ProQuest). Drivers and response variables are listed in Table 1.

**Table 1.** Drivers and response variables included in the systematic review.

Drivers	Biodiversity response variables
Spatial crop diversity	Species extinction
Temporal crop diversity	Extinction risk
Crop type	Species richness
	Abundance
	Community composition
	Assemblage
	Rare species richness
	Rare species abundance
	Relative species rarity

This review is global and no geographical limitations will be used. Both peer-reviewed and grey literature databases will also be included to minimise publication bias and increase the comprehensiveness of the review. For feasibility and with reference to differences in orders of magnitude found in nature, included studies will measure biodiversity among two or more species of flora or invertebrate fauna; or one or more species of vertebrate fauna.

*Inclusion criteria:*

- Full-text articles in English
- Controlled experiments or observational studies
- Quantitative studies on the impacts of crop diversity or crop type on one of the following biodiversity metrics: extinction, extinction risk, species richness, population abundance, assemblage, community composition, rare species richness/abundance or relative rarity
- Response variables measure two or more species of plants or invertebrate fauna; or one or more species of vertebrate fauna
- Drivers measure crops grown or cultivated for immediate human or livestock use or consumption including food, feed, fibres, fuels, and grasslands for pasture/grazing
- Crop diversity measures include mixed crop-livestock or crop-aquaculture systems
- Publication years: 1990-present

The following controls/reference habitats will be included:

- Spatial crop diversity (mixed, pattern or inter-cropping) compared to monoculture
- Spatial crop diversity (field or plot-level diversity) at farm or landscape scale compared to greatest crop homogeneity/least crop heterogeneity
- Temporal crop diversity (crop rotation) compared to lack of rotation (i.e. single crop continuously cultivated)
- Crop types compared to other crop types

*Exclusion criteria:*

- Review articles with no original results presented
- Simulation or scenario-based modelling studies
- Qualitative studies
- Studies with biodiversity response variables measuring:
  - seeds or seed banks;
  - larvae or eggs;
  - genetic, phylogenetic, or functional biodiversity
- Studies with observations from non-agricultural areas e.g. natural/semi-natural margins, hedgerows, banks, ditches, meadows, grasslands, forests, copses, woods, riparian lands, wetlands or other landscape features. This includes plants/trees that are managed but not on agricultural land (e.g. poplar managed as a riparian woodland)
- Studies on damaged, contaminated, or disturbed land or on land restored or reclaimed from damage caused by natural or anthropogenic disturbances e.g. fire, soil salinity, heavy metals, previous high-intensity agriculture
- Studies within ponds, streams or river habitats
- Before-after studies without controls/reference habitats; land use history or legacy effects studies
- Studies comparing different grassland mixes to one another
- Agricultural management practices (e.g. tillage, mulching, fallow, use of fertilisers or pesticides) applied inequitably to the intervention/driver study group and the control/reference study group and unadjusted for in estimates of effect
- Crop diversity:
  - Diversity measures include genetic modification, varieties, or cultivars
  - Field-level diversity measures include non-crop plants, herbs, covers, shrubs or trees grown for soil or ecological health but not cultivated as a marketable crop for immediate human or livestock use (e.g. agroforestry inclusive of non-crop plants/trees, shaded coffee, shaded cocoa, non-crop “covers”, green manures)
- The following controls/reference habitats will be excluded:
  - Monoculture or continuous cultivation of a crop which is not included within the crop diversity study group
  - Crop type: natural grazing lands e.g. meadow, rangeland

A set of complete search terms for the Web of Science database is available as extended data ^22^. Key concepts are captured by three topics: 1) crop diversity, 2) crop type and 3) biodiversity metrics. Use of “Near/15” will link exposure-related terms to agricultural landscapes, while “Near/5” specifies precise exposure and outcome terms observed in the literature and close variants thereof. In addition to terms identified in preliminary searches, the Food and Agriculture Organization (FAO) Indicative Crop Classification (ICC) was used to help construct the crop type search terms ^23^, and the BIOSIS Citation Index list of taxa notes were used to help construct the list of biodiversity search terms ^24^. The search strategy has been reviewed by an experienced librarian with no other collaboration on the project.

### 3.2. Screening, data coding, and data extraction

To screen and extract data, search results will first be downloaded to an Endnote database. Duplicates will be removed, first electronically (exact match only), then manually to account for misspellings and slight differences. Titles/abstracts will first be screened for inclusion and exclusion criteria and then full text papers (CM). A second independent reviewer will screen 10% of titles/abstracts and full texts. Discrepancies will be discussed and agreed by consensus, with a third reviewer if necessary (FH). Data will be coded and extracted by a member of the review team (FB); the primary reviewer (CM) will independently assess coding and data extracted for 10% of full texts included. For the papers identified for inclusion in the review, data coded and extracted will include the following: authors, year, publication, study location, study design, scale, biodiversity metric, species/taxa (super taxa, taxa, organism classifier, organism name), crop type, crop diversity, duration of intervention, number of crop rotations, effect sizes, standard deviations, sample sizes, biome, ecoregion, climatic zone, field size, and other agricultural management, landscape, environmental and climatological factors.

### 3.3. Data management

Search results including titles and abstracts will be exported to and managed within Endnote. Complete results for each database will be maintained, as will duplicates excluded and the results of each stage of screening. Papers including in the titles/abstracts screening stage will be moved to EPPI Reviewer for full text review. Full texts reviewed and excluded will be categorised by reason for exclusion with notes maintained using the designated field in the EPPI Reviewer record. If a full text and/or data is not available directly in the text, the corresponding author will be contacted and up to two contact efforts will be made. A contact record sheet will be kept with author names and study title, email addresses, dates(s) of contact, and results of contact. If no new contact information can be identified and there is no response from the author, or if the author declines to share data, the study will be excluded from further analysis. This will be noted in the study limitations in the final review report.

A pilot data coding and extraction form will be developed at the outset of the data extraction process. This will be used to create a set of hierarchical codes within EPPI Reviewer. Data from the first five full text papers included in the review will be extracted using the codes. They will then be adapted as needed to best reflect common data formats and data re-extracted as required from the first five papers. This process will be repeated until no further adaptation is required. Each form with data extracted will be exported and dated for tracking.

### 3.4. Study quality and risk of bias assessment

Adapting the quality assessment tool developed by the Critical Appraisal Skills Programme (CASP) ^25^, the following questions will be used to assess each study meeting the full inclusion criteria:

- Was there a clear description of the crops evaluated?
- Was there a clear description of the biodiversity metrics evaluated?
- Was there a clear description of the species and taxa evaluated?
- Was a clear description given of field conditions and agricultural practices used?
- Was a clear justification given for conducting a study in a particular area – including a description of agricultural conditions?
- Were crops under the “intervention” compared to an appropriate and comparable baseline group or situation?
- Were the methods of measuring the agricultural exposure(s) clearly described?
- Were the methods of measuring the biodiversity outcome(s) clearly described?
- Are sufficient data presented to support the findings?
- Were analyses described in detail?
- Did the researchers critically examine their potential biases during measurement, analysis and selection of data for presentation?

Papers will be scored between 1–11, with 1 mark given for each ‘Yes’ above. To assess risk of bias, the Environmental-Risk of Bias tool will be adapted and a low, high or unclear mark will be given for each of the following categories: selection bias, performance bias, detection bias, attrition bias, reporting bias and other bias related to study design ^26^. Quality and risk of bias assessment results will be reported for all papers, and any papers scoring less than 8 and/or presenting insufficient data to support the findings will be excluded from further synthesis. The quality assessment review will be done by a member of the review team (FB) and the primary reviewer (CM) will independently assess 10% of the full texts included.

### 3.5. Data synthesis

Data synthesis will aim to explore both patterns and dispersion in the data. It will first be conducted using the following three steps: 1) complete a textual description of studies, 2) tabulation of studies by groups and clusters, and 3) preliminary synthesis and development of a common results rubric. To tabulate studies, results will be grouped by 1) biodiversity metric, followed by 2) driver, 3) species/taxa, and 4) control/reference habitat. Species/taxa may be combined where appropriate up to the super taxa level e.g. ants and spiders re-categorised as arthropods. Measures of exposure such as all-crop diversity (e.g. over both space and time) or crop type by vegetation structure (e.g. orchard crops) may also be grouped subject to similarity of the comparison groups.

*Evidence mapping and narrative synthesis*

Results for certain data groups (exposures: crop type; outcomes: extinction, extinction risk, assemblage, relative rarity) may be insufficient in number and/or highly heterogeneous. Therefore quantitative synthesis will be infeasible or unlikely to be robust. In such event, results will be described by heat map, identifying the number of studies providing evidence by outcome, exposure and taxa or super taxa (population). If results are of a sufficient number but highly heterogeneous, thematic analysis will be conducted using narrative approaches and finally, conceptual mapping will be conducted to explore relationships between the findings.

*Quantitative analysis*

Three outcomes will be considered for quantitative analysis: species richness, abundance, and the Shannon’s diversity index as these metrics tend to be those most often measured. By taxa category, statistical summary will be explored if there are a sufficient number of study results which also report the effects of the same exposure. Further criteria for statistical summary will include use of experimental and observational study designs and availability of variance estimates and sample sizes. All data from the extraction form will be imported for handling into the R environment. RStudio 4.0.0 is a free software environment for statistical computing and graphics ^27^. Using the R package metafor (version 2.4.0), effect sizes for species richness and abundance will be calculated as response ratios (the magnitude of difference between groups), which do not require measures of within-group variance and are commonly used in the ecological sciences because results from different study designs, scale and taxonomic groups may be appropriately combined ^28^. Random effects meta-analysis models will also be used to account for heterogeneity and study identifier will be set as the random effect. If present in a sufficient number of studies, agricultural management covariates will also be included in the models. The estimated range of true effects i.e. differences in effects observed, will be reported using forest plots and confidence intervals. Sensitivity analyses will also be conducted by comparing results of full models with those: 1) without observational studies and 2) of low study quality (defined as a score of <9 marks after following the procedure outlined in section 3.4).

Data synthesis will be conducted by the first author (CM) and reviewed by other contributors.

## 4. Sources of bias

Reviewer bias: Inclusion and exclusion criteria may be interpreted differently. A third reviewer will be identified if discrepancies arise between the first two reviewers.

Publication bias: If statistical summary is conducted, Rosenthal’s fail safe number – the number of unpublished studies reporting no evidence of effects that would need to be added to a summary analysis in order to change the results – will be calculated to indicate the credibility of the results. If this is infeasible due to study heterogeneity, then lack of ability to estimate publication bias will be acknowledged as a limitation of the study in the final reporting.

Selective reporting bias: Because it is not common practice in the environmental sciences to register experimental study protocols prospectively, it is not possible to evaluate within-study selective reporting. This limitation will be acknowledged in the final systematic review report.

Inconsistent outcome definitions and methods: There are differences in the way that biodiversity metrics (e.g. relatively rarity) are measured, defined or calculated by ecological researchers. Differences will be carefully considered prior to data synthesis.

## 5. Outputs

Results of the analysis will map and/or synthesise evidence of the effects of crop diversity and crop type on a variety of different taxa and biodiversity metrics. Gaps in the literature will also be identified, with a view toward future research and policy directions for nutrition, food systems and environment. Key outputs from the systematic review will include a full literature database on the effects of crop diversification and crop type on biodiversity, tables of study characteristics and of synthesised analyses and/or evidence map and narrative summarising results.

## 6. Ethics and dissemination

This review will not use data collected from human subjects. An application for ethical approval has been approved by the London School of Hygiene & Tropical Medicine Ethics Committee (ref 17546). Findings will be published in a peer-reviewed journal.

## 7. Study status

The study protocol and search strategy have been completed; as of publication, searching was completed in August 2019. Screening of titles/abstracts was completed as of May 2020. Full text screening was completed as of July 2020.

## 8. Data availability

### Underlying data

No data is associated with this article.

### Extended data

Figshare: Extended Data File 1 Search Terms.docx. https://doi.org/10.6084/m9.figshare.8290004.v1 ^22^

This project contains the following extended data:

- Extended Data File 1 Search Terms.docx (Web of Science BIOSIS Citation Index systematic review search terms)

### Reporting guidelines

Figshare: Completed PRISMA-P checklist for ‘The effects of crop diversity and crop species on biological diversity in agricultural landscapes: a systematic review protocol’. https://doi.org/10.6084/m9.figshare.8290088.v1 ^29^

Data are available under the terms of the Creative Commons Zero "No rights reserved" data waiver (CC0 1.0 Public domain dedication).
