# Supplementary material for: The effects of crop diversity and crop type on biological diversity in agricultural landscapes: a systematic review protocol
Source: Wellcome Open Res. 2020 Jul 27;4:101. Originally published 2019 Jun 26. [Version 2] doi: 10.12688/wellcomeopenres.15343.2 (PMC7241271; doi:10.12688/wellcomeopenres.15343.2)
Supplement: Peer review history

# Peer review: Reviewer response for version 1

**DOI:** 10.21956/wellcomeopenres.16753.r38200

**Published:** 2020-05-20

**Authors:** Todd Rosenstock

**Affiliations:** 1 World Agroforestry (ICRAF), Nairobi, Kenya

The systematic review would examine two principal questions (1) whether increased crop diversity, either spatial (e.g., intercropping) or temporal (e.g., crop rotations) affect biodiversity including flora, fauna, and microbes, namely bacteria, fungi, algae, and protozoa and (2) whether crop species also affect biodiversity. These questions are of high interest to researchers and decision-makers. A few general comments to consider below.

 Please consider being more specific (and perhaps narrowing) about the agronomic practices - biodiversity outcomes that will be investigated. There are a number of syntheses available on rotations and intercrops on microbial diversity, earthworms, insect populations, etc. (see references included with the review for a few). It may be important to consider how the proposed review fits within and adds to this constellation of existing reviews, amongst other papers not mentioned. 

 Coding rotations and intercrops in meta-analysis is often a problem because of the large diversity of species mixtures and variation in the comparators. This translates to very difficult (and sometimes meaningless) comparisons agronomically. When comparisons are valid, effects can also be confounded by other management aspects (how residues are handled mulched, incorporated, burned, moved off-farm, use of agrochemical or not, etc). This is likely to require many iterations during the review.

 Consider reducing the outcome indicators to narrow the question. The review may be very large and difficult to manage when speaking about flora, fauna, and microbes together. The initial feeling is that the first question is already a very large undertaking and perhaps valuable to focus on only one of the two questions (though they are related).

 Please clarify the mechanism implied in the sentence, "Understanding the relationships between crop species and biodiversity..." in the 3rd paragraph of the background. This seems to be a key justification for the 2nd question of the review. But the implications of potential findings are less clear.

 Is the question about biodiversity writ large or functional diversity? These are often highly managed systems. 

 Consider looking toward the agricultural ontologies to help further refine search terms. The CGIAR Big Data Platform has a working group on agricultural ontologies and links to the sources. This will also ensure interoperability.

 Inclusion criteria. Suggest to only used controlled experiments. Otherwise, you may find a lot of noise in the dataset. Consider focusing on a more limited number of cropping systems or farming systems and then expanding based on success. 

 Does rotation include green manures grown between seasons?

 Please clarify if agroforestry is considered spatial crop diversity.

 Will there be any quality control on the sampling and measurements used or the ways in which practices are implemented that will warrant exclusion?

 Overall, the protocol is clearly written and suggests a high degree of rigor. The primary challenge will be the expansive scope and coding very heterogeneous management and outcomes. I look forward to seeing what comes up.

Is the study design appropriate for the research question?

Yes

Is the rationale for, and objectives of, the study clearly described?

Partly

Are sufficient details of the methods provided to allow replication by others?

Yes

Are the datasets clearly presented in a useable and accessible format?

Not applicable

Reviewer Expertise:

agriculture and ecology

I confirm that I have read this submission and believe that I have an appropriate level of expertise to confirm that it is of an acceptable scientific standard, however I have significant reservations, as outlined above.

## References

[1] : The impact of crop rotation on soil microbial diversity: A meta-analysis. *Pedobiologia*.2016;59(4) : 10.1016/j.pedobi.2016.04.001 215-223 10.1016/j.pedobi.2016.04.001

[2] : Ecological intensification and arbuscular mycorrhizas: a meta-analysis of tillage and cover crop effects. *Journal of Applied Ecology*.2017;54(6) : 10.1111/1365-2664.12815 1785-1793 10.1111/1365-2664.12815

[3] : Plant diversity effects on soil microorganisms: Spatial and temporal heterogeneity of plant inputs increase soil biodiversity. *Pedobiologia*.2016;59(4) : 10.1016/j.pedobi.2016.04.004 175-177 10.1016/j.pedobi.2016.04.004

[4] : Managed ecosystems: biodiversity and ecosystem functions in landscapes modified by human use.2009; 10.1093/acprof:oso/9780199547951.003.0013 178-194 10.1093/acprof:oso/9780199547951.003.0013

[5] : A global synthesis reveals biodiversity-mediated benefits for crop production. *Sci Adv*.5(10) : 10.1126/sciadv.aax0121 eaax0121 10.1126/sciadv.aax0121 31663019PMC6795509

[6] : Do cover crops benefit soil microbiome? A meta-analysis of current research. *Soil Biology and Biochemistry*.2020;142: 10.1016/j.soilbio.2019.107701 10.1016/j.soilbio.2019.107701

[7] : Increasing crop diversity increased soil microbial activity, nitrogen-sourcing and crop nitrogen, but not soil microbial diversity. *South African Journal of Plant and Soil*.2017;34(5) : 10.1080/02571862.2017.1317852 371-378 10.1080/02571862.2017.1317852

[8] : Long-Term Evidence Shows that Crop-Rotation Diversification Increases Agricultural Resilience to Adverse Growing Conditions in North America. *One Earth*.2020;2(3) : 10.1016/j.oneear.2020.02.007 284-293 10.1016/j.oneear.2020.02.007

[9] : Intercropping with legume for agroecological cropping systems: Complementarity and facilitation processes and the importance of soil microorganisms. A review. *Agriculture, Ecosystems & Environment*.2017;240: 10.1016/j.agee.2017.02.019 148-161 10.1016/j.agee.2017.02.019

[10] : Meta-analysis shows positive effects of plant diversity on microbial biomass and respiration. *Nature Communications*.2019;10(1) : 10.1038/s41467-019-09258-y 10.1038/s41467-019-09258-y PMC643080130902971

[11] : Effects of agroforestry on pest, disease and weed control: A meta-analysis. *Basic and Applied Ecology*.2015;16(7) : 10.1016/j.baae.2015.08.006 573-582 10.1016/j.baae.2015.08.006

[12] : A global meta-analysis of the biodiversity and ecosystem service benefits of coffee and cacao agroforestry. *Agriculture, Ecosystems & Environment*.2013;175: 10.1016/j.agee.2013.05.003 1-7 10.1016/j.agee.2013.05.003

# Peer review: Reviewer response for version 1

**DOI:** 10.21956/wellcomeopenres.16753.r38204

**Published:** 2020-04-15

**Authors:** Matteo Dainese

**Affiliations:** 1 Institute for Alpine Environment, Eurac Research, Bolzano, Italy

This study protocol provides a clear methodological framework to conduct a synthesis literature on the effects of spatial and temporal crop diversity and crop species on biodiversity in agricultural landscapes. Thank you for the opportunity to review this interesting article. The protocol is very ambitious, well described and will provide very important results.

 I have some small comments for the authors.

**Background**

- Crop diversification at the landscape level is another spatial dimension that might be considered in this synthesis. Evenness is another important biodiversity attribute. Will you consider it?  

**Aim and objectives**

- Another secondary question could be: Do different biodiversity metrics respond equally to crop diversity?

**Methods**

- I would spend some more words to explain Table 1. What do you mean with species extinction and extinction risk in this context? Which metric will you consider to measure these biodiversity outcomes?
- I have also some concern about the use of ‘exposure’ and ‘outcome’ terms in this context. They are uncommon in ecological studies.
- Which grey literature databases will you consider?
- How will you consider the different biodiversity metrics and methodologies from different studies?
- Testing the causal pathways by which crop diversity or crop species may have effects on biodiversity is one of questions that this study seeks to answer. How do you think to address this point?

Is the study design appropriate for the research question?

Yes

Is the rationale for, and objectives of, the study clearly described?

Yes

Are sufficient details of the methods provided to allow replication by others?

Yes

Are the datasets clearly presented in a useable and accessible format?

Not applicable

Reviewer Expertise:

Agroecology - Biodiversity

I confirm that I have read this submission and believe that I have an appropriate level of expertise to confirm that it is of an acceptable scientific standard.

# Peer review: Reviewer response for version 1

**DOI:** 10.21956/wellcomeopenres.16753.r35846

**Published:** 2019-08-30

**Authors:** Sarah Redlich

**Affiliations:** 1 Department of Animal Ecology and Tropical Biology, Biocenter, University of Würzburg, Würzburg, Germany

**General comments**

 Biodiversity loss across all taxa and scales is a hugely important topic, especially in the light of global change, ecosystem resilience and resistance. At the same time, agricultural intensification plays a major role in species declines, necessitating a move towards biodiversity-friendly farming practices. The value of crop diversity and crop type for soil quality and productivity has long been shown, and numerous studies and literature reviews highlight the benefits for biodiversity as well. However, most of these papers concentrate on specific taxonomic groups or study systems, and quantitative syntheses of crop diversity effects are mainly lacking. For instance, two published quantitative reviews by Dassou & Tixier (2016) ^1^ and Letourneau *et al*. (2011) ^2^ focus primarily on abundance and/or richness measures of herbivores and predators, ignoring other taxonomic groups. Therefore, quantitatively summarising the effects of crop diversity and crop type on different taxa and other aspects of biodiversity such as extinction risk is an essential step forward.

 This study protocol proposes a method to screen and synthesise literature related to the benefits of crop diversity and crop species on biodiversity in agricultural landscapes. The authors propose to review both peer-reviewed and grey literature databases using a variety of different search terms, then applying thorough quality and bias assessment of potential studies, before narratively and quantitatively synthesising the effects.

 While acknowledging the great importance of the proposed review, there are a few general shortcomings of the study protocol that I would like to address.

 Next to temporal crop diversity (crop rotations), the authors mention mixed cropping or intercropping as a type of spatial crop diversity included in the review. What about crop diversity on a landscape scale, i.e. not on the same field? This spatial crop diversity can have positive effects on biodiversity and ecosystem services (e.g. Fahrig *et al*. 2015 ^3^; Hiron *et al*., 2015 ^4^; Palmu, Ekroos, Hanson, Smith, & Hedlund, 2014 ^5^; Redlich, Martin, & Steffan‐Dewenter, 2018 ^6^). It also plays an important role in agricultural policy, for example in Europe, where farmers are obliged to increase the number of crops grown on a farm.

 On the other hand, I slightly struggle with the term ‘crop species’ used throughout the paper. For two reasons:

 First, I think that the term ‘crop species’ is slightly misleading in this context, as it could also imply the richness of crop species, which is obviously not the intention. Using a term such as ‘crop type’ or ‘crop identity’ may be helpful.

 Second, quantitatively assessing the effect of ‘crop species’ is non-trivial, because there are numerous crops grown worldwide (and there is no geographical restriction applied in this review), and each crop could have been compared to numerous other crops or mixed natural-agricultural systems. These different crop-crop combinations, however, can only be assessed with difficulty and a lot of effort, and most likely not using quantitative measures. Rightly, the authors expect this issue and propose narrative and mapping approaches instead. They also suggest the grouping of crops whenever needed or possible (e.g. by crop characteristics/functions). To me, the latter approach is most valid and useful, as different studies have shown the benefits of using functional groupings over crop species per se. In this case, however, the title and use of the term ‘crop species’ is misleading.

 As a last general comment, the terms “exposure” and “outcome” are not normally used in ecology (which is the primary field of research this review focuses on) to describe drivers (crop diversity and crop type) and response variables (biodiversity metrics). In ecological studies, the ‘outcome’ would be that effects are either positive, neutral or negative for biodiversity.

 Apart from these and some minor points (see below) that may require some clarification, I recognize the value of the planned review and the generally strong and thorough design of the study protocol. I am very much looking forward to seeing some first results of a quantitative synthesis of crop diversity effects on biodiversity.

**Section-specific comments**

Abstract

 I missed a sentence about why the loss of biodiversity is considered a problem, especially in agricultural systems.

  “Crop species are also known to have independent impacts on biodiversity.” This sentence is not very clear, especially the meaning of ‘crop species’. I guess the authors aim to say that depending on the crop type, effects on biodiversity can differ (independently of overall crop diversity benefits).

 “… and a proportion will be independently conducted by a second reviewer.” The idea that the second reviewer takes over the task of quality control only becomes clear when reading the methods section.

Background

 I missed the link between land use (which can include a lot of changes not only related to agriculture) and agricultural intensification.

 “These factors, together with crop species and related management and production cycles, determine the intensity of agricultural management”. As in the abstract, the exact meaning of ‘crop species’ in this context is not clear to me. I assume the authors mean to say that the type of crop grown also determines the management practices and crop rotations required, and therefore makes farming more or less intensive (e.g. oilseed rape farming requires high insecticide inputs, while winter wheat needs large amounts of fertilizer inputs and is often grown in short rotations).

 I agree that land use and land use change affect biodiversity, but not necessarily why “these factors” should determine the intensity of agricultural management, unless a change in land use involves growing more management intensive crops, monocultures etc.

 What is the difference between ‘rotation of crops’ and ‘the practice of growing different crops in the same field, rotated seasonally or annually'?

Aim and objectives

 Secondary questions and study objectives are redundant (e.g. “Are there trends in the response of biodiversity to crop diversity across different taxonomic groups or biomes?” and “To identify trends in the response of biodiversity to crop diversity across different taxonomic groups or biomes”). I recommend focussing on study objectives, as these more clear.

Methods

 Methods, statistical analyses and visualisation options seem to be appropriate for the purpose of this review.

 I very much like the fact that the literature search is not restricted to scientific literature, but also grey literature and dissertations/thesis. While some may argue that the quality of such literature may not compare to peer-review research articled, I am a strong advocate for recognizing the value of research that does not end up being published in scientific journals, as an abundant amount of relevant evidence otherwise gets lost. I also believe that applying quality and bias assessment as described later will ensure comparable standards for both peer-reviewed and grey literature.

 The list of databases and the search terms used to find papers assessing drivers and biodiversity metrics seem to be quite comprehensive. The authors make huge efforts to double-check the screening and coding process, ensure the study quality and reduce the risk of methodological/design/publication bias. Notwithstanding my comments above about pooling crop species, using narrative description and mapping would be a nice (but time-consuming) way to deal with insufficient/heterogeneous data. I also very much appreciate the protocol for recoding every step of the review process and data acquisition, as well as applying the Critical Appraisal Skills Programme used in healthcare science to an ecological study!

 Some other comments:

- For biodiversity metrics: I suggest to stick with the same term and order in the table and text (e.g. always “relative species rarity”).
- What does the inclusion criterion ‘All years’ refer to?
- “comparators” does not sound like the right word in this context. “Control” or even “reference/baseline crop/habitat” may be more suitable.
- Again, what about across-field spatial crop diversity? In this case the control would be a landscape with low spatial crop diversity (e.g. multi-crop landscapes compared to landscapes with only a few crops grown).
- “Exposure effects presented solely in combination with landscape composition or other agricultural management effects e.g. non-crop vegetation or structures (except grasslands used for pasture/grazing), no-till, etc” This is not clear to me.
- Options to shorten and combine: “A contact record sheet will be kept with author names and study title, email addresses, dates(s) of contact, and results of contact. If no new contact information can be identified and there is no response from the author, or if the author declines to share data, the study will be excluded from further analysis”.

Is the study design appropriate for the research question?

Yes

Is the rationale for, and objectives of, the study clearly described?

Yes

Are sufficient details of the methods provided to allow replication by others?

Yes

Are the datasets clearly presented in a useable and accessible format?

Not applicable

Reviewer Expertise:

Agroecology, ecosystem services and biodiversity.

I confirm that I have read this submission and believe that I have an appropriate level of expertise to confirm that it is of an acceptable scientific standard, however I have significant reservations, as outlined above.

## References

[1] : Response of pest control by generalist predators to local-scale plant diversity: a meta-analysis. *Ecol Evol*.6(4) : 10.1002/ece3.1917 1143-53 10.1002/ece3.1917 26839684PMC4725331

[2] : Does plant diversity benefit agroecosystems? A synthetic review. *Ecological Applications*.2011;21(1) : 10.1890/09-2026.1 9-21 10.1890/09-2026.1 21516884

[3] : Farmlands with smaller crop fields have higher within-field biodiversity. *Agriculture, Ecosystems & Environment*.2015;200: 10.1016/j.agee.2014.11.018 219-234 10.1016/j.agee.2014.11.018

[4] : The relationship of bird diversity to crop and non-crop heterogeneity in agricultural landscapes. *Landscape Ecology*.2015;30(10) : 10.1007/s10980-015-0226-0 2001-2013 10.1007/s10980-015-0226-0

[5] : Landscape-scale crop diversity interacts with local management to determine ground beetle diversity. *Basic and Applied Ecology*.2014;15(3) : 10.1016/j.baae.2014.03.001 241-249 10.1016/j.baae.2014.03.001

[6] : Landscape-level crop diversity benefits biological pest control. *Journal of Applied Ecology*.2018;55(5) : 10.1111/1365-2664.13126 2419-2428 10.1111/1365-2664.13126
